# Four new species and two new records of genus *Zeugophora* (Coleoptera, Megalopodidae, Zeugophorinae) from China

**DOI:** 10.3897/zookeys.975.53472

**Published:** 2020-10-12

**Authors:** Kai-Qin Li, Hong-Bin Liang

**Affiliations:** 1 Kunming Natural History Museum of Zoology, Kunming Institute of Zoology, Chinese Academy of Sciences, Kunming 650223, China Chinese Academy of Sciences Kunming China; 2 Key Laboratory of Zoological Systematics and Evolution, Institute of Zoology, Chinese Academy of Sciences, Beijing 100101, China Chinese Academy of Sciences Beijing China

**Keywords:** Celastraceae, new record, new species, *
Pedrillia
*, *
Zeugophora
*, Zeugophorinae

## Abstract

Four new species of the subgenus Pedrillia Westwood, 1864 are described from southwest and central China: Zeugophora (Pedrillia) euonymorum**sp. nov.**, Zeugophora (Pedrillia) flavithorax**sp. nov.**, Zeugophora (Pedrillia) trifasciata**sp. nov.** and Zeugophora (Pedrillia) yuae**sp. nov.** Two species are recorded for the first time in China and redescribed: Zeugophora (Zeugophora) turneri Power, 1863 and Zeugophora (Pedrillia) nigricollis Jacoby, 1885. To date, a total of 35 Zeugophorinae species has been recognized in China.

## Introduction

The genus *Zeugophora* Kunze, 1818 belongs to the subfamily Zeugophorinae in the family Megalopodidae. A total of 29 species of the subfamily Zeugophorinae has been recorded in China (Lee and Cheng 2007; Li and Liang 2018). The Chinese fauna consists of *Zeugophora* with two subgenera *Pedrillia* Westwood, 1864 and *Zeugophora* Kunze, 1818. However, the status of *Pedrillia* Westwood is controversial, and it has been treated as a distinct genus by some authors (Baly 1873; Jacoby 1908; Chûjô 1935, 1937; Tan et al. 1980; Yu and Yang 1997), or as a subgenus of *Zeugophora* (Crowson 1946; Monrós 1959; Gressitt and Kimoto 1961; Chen and Pu 1962; Kimoto and Gressitt 1979; Medvedev 1985, 1997; Reid 1989, 1992, 1995; Schöller 2009; Warchałowski 2010), or even a synonym of *Zeugophora* (Bryant 1943; Sekerka and Vives 2013). In the present paper, we treat *Pedrillia* as a subgenus as we did previously (Li and Liang 2018).

In recent years, a number of expeditions have been made for collecting specimens of *Zeugophora*. On Songshan Mountain near Beijing City, a quite large number of *Zeugophora* were collected using Malaise traps, a tool which was originally used to collect wasps and flies. In Ningxia and Guizhou, *Zeugophora* specimens were collected by beating plants in the family Celastraceae. In the museum of the Kunming Institute of Zoology, two specimens of *Zeugophora* were sorted out from samples collected for ecological monitoring. After carefully comparison with types and named specimens, we found four species new to science and two records new to China from said specimens. Their descriptions are given below.

## Materials and methods

**Abbreviations**:

**KIZ**Kunming Natural History Museum of Zoology, Kunming Institute of Zoology, Chinese Academy of Sciences, Kunming, China.

**IZCAS**National Zoological Museum of China, Institute of Zoology, Chinese Academy of Sciences, Beijing, China.

**NHMUK**The Natural History Museum, London, UK.

### Species studied

Thirteen species belonging to one genus and two subgenera of Zeugophorinae were studied and examined (Table 1). All specimens studied in this paper are deposited in KIZ unless otherwise stated.

**Table 1.** Species of Zeugophorinae studied and examined.

Genus (Subgenus)	Species	Number of specimens	Depository
Zeugophora (Zeugophora)	*turneri* Power, 1863	61	IZCAS, KIZ
	*cribrata* Chen, 1974	8	IZCAS
	*cyanea* Chen, 1974	16	IZCAS
	*scutellaris* Suffrian, 1840	15	IZCAS
Zeugophora (Pedrillia) bbe	*annulata* Baly, 1873	25	IZCAS, KIZ
	*bicolor* Kraatz, 1879	6	IZCAS
	*euonymorum* Li & Liang, sp. nov.	24	IZCAS, KIZ
	*flavithorax* Li & Liang, sp. nov.	3	KIZ
	*maculata* (Chûjô, 1941)	8	KIZ
	*nigricollis* Jacoby, 1885	2	KIZ
	*tricolor* Chen & Pu, 1962	5	IZCAS, KIZ
	*trifasciata* Li & Liang, sp. nov.	2	KIZ
	*yuae* Li & Liang, sp. nov.	8	IZCAS, KIZ

### Methods

Dry specimens were soaked in boiled water for 1–2 hours. The lateral margin of the abdomen was opened and the genitalia were pulled out of the abdomen with fine forceps, or the whole abdomen was removed from the specimen. The genitalia or abdomen were soaked in a warm solution of 10% KOH or NaOH for 10–20 minutes as a treatment. The treatment time depended upon the degree of sclerotization of the genitalia in different species. After treatment, these organs were washed with water, then dyed with Chlorazol Black E for one second. The genitalia were then detached and transferred to glycerin for observation, photography, and preservation.

All measurements were made using a Nikon SMZ1500 or a Nikon SMZ18 stereoscopic microscope with the aid of an ocular micrometer. Body length (BL) = the linear distance along the midline from the anterior margin of the labrum to the apex of the elytra; body width (BW) = elytra width (EW) = the maximum linear distance across the elytra; pronotum length (PL) = the linear distance along the median line of the pronotum; pronotum width (PW) = the linear distance across the widest part of the pronotum; pronotum width at the apex (PAW) = the linear distance across the apex of the pronotum; pronotum width at the base (PBW) = the linear distance across the base of the pronotum; elytra length (EL) = the linear distance from the base of the elytra to the apex of the sutural angle; median lobe length = the linear distance from the base to the apex; median struts length = the linear distance from the base to the apex. Ratios cited in descriptions are based on these measurements.

Photographs of male genitalia were taken using a Nikon SMZ-1500 stereoscopic dissecting microscope fitted with a Cannon 450D digital camera or Nikon SMZ18 stereoscopic dissecting microscope fitted with a Nikon D610 digital camera. For each final image, several photographs were taken using different focal planes, combined with Helicon Focus software to obtain one synthesized photograph, and finally edited with Adobe Photoshop software.

Morphological terminology for male genitalia follows Snodgrass (1935), Verma (1996), Chûjô (1952, 1953), Kasap and Crowson (1985), Li and Liang (2018), and Li et al. (2013).

## Taxonomy

### Genus *Zeugophora* Kunze, 1818

#### Subgenus Zeugophora Kunze, 1818

##### Zeugophora (Zeugophora) turneri

Taxon classificationAnimaliaColeopteraMegalopodidae

Power, 1863 new record

232B4D5A-770C-5F29-ACE1-16E79A9DFA09

Figures 1–6 , 7–11

Zeugophora turneri Power, 1863: 8735.
Zeugophora rufotestacea Kraatz, 1871: 162.

###### Specimens examined.

Sixty-one males and females (IZCAS, KIZ), China, Beijing, Songshan, Haituoshan, Xiaohunpo, 1100 m, 2013.ix.14, Bo Liu coll.

###### Diagnosis.

Antennae, head, pronotum, scutellum, elytra, prosternum and legs yellowish brown; apex of mandible black, mesoventrite, metaventrite and abdominal ventrites black; dorso-central portion of median lobe with a slender sclerite, apex of median lobe gradually narrowed.

**Figures 1–6.**
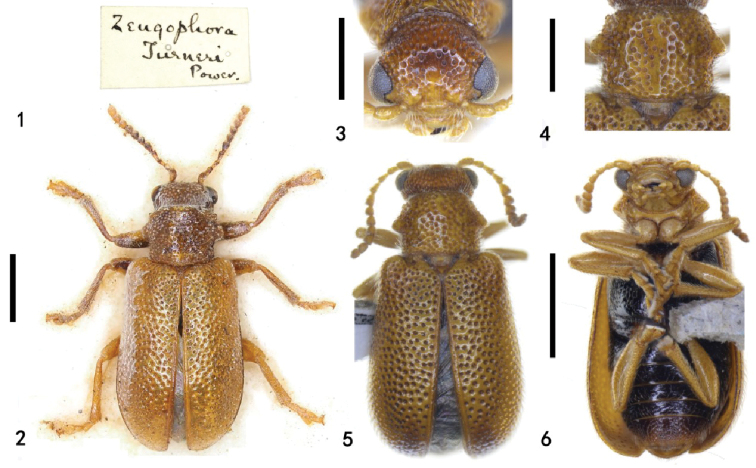
*Zeugophora turneri* Power. **1, 2** specimen identified by Power **1** label **2** dorsal view **3, 4** common specimen from Beijing **3** head, anterior view **4** pronotum, dorsal view **5** dorsal view **6** ventral view. Scale bars: 1.0 mm (**1, 5, 6**); 0.5 mm (**3, 4**).

**Figures 7–11.**
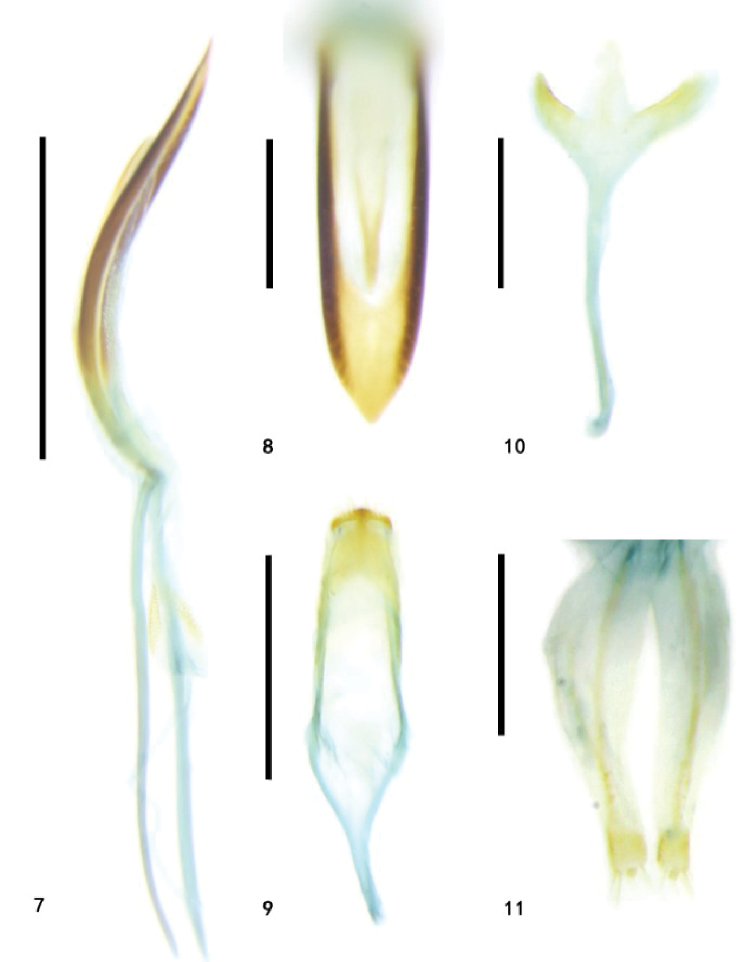
Genitalia of *Zeugophora turneri* Power, genitalia **7–10** male genitalia **7** median lobe and median struts, lateral view **8** median lobe, dorsal view **9** tegmen, dorsal view **10** spiculum, dorsal view **11** ovipositor, dorsal view. Scale bars: 0.5 mm (**7, 9**); 0.2 mm (**8, 10, 11**).

###### Description.

BL = 3.0–3.2 mm, BW = 1.4–1.5 mm. Antennae, head, pronotum, scutellum, elytra, prosternum and legs yellowish brown; apex of mandible black, mesoventrite, metaventrite and abdominal ventrites black.

**Head**: eyes prominent, inner margin with shallow canthus; center of vertex without puncture and pubescence, sides of vertex coarsely punctate and pubescent; occiput constricted, densely punctate and pubescent; frons coarsely punctate and pubescent, center concave; clypeus trapezoid, anterior margin and lateral sides with punctures and pubescence; fronto-clypeal suture prominently backwards in the center; labrum rectangular, 3 × as long as wide; antennae short, extending to the humeri, antennomere 1 swollen, antennomere 2 slightly shorter than antennomere 1, antennomere 3 as long as antennomere 1, antennomere 4 as long as antennomere 3, antennomeres 5–11 short and broad, equal in length, as long as antennomere 2, apex of antennomere 11 acute.

**Thorax**: PW/PL = 1.3–1.5; anterior margin slightly flattened, posterior margin arching backwards in the center, length of anterior margin nearly equal to posterior margin; anterior margin indistinct; posterior margin backwards medially; anterior and posterior groove indistinct; lateral margins subparallel at anterior portions, lateral tubercle prominent laterally, blunt at apex; disc slightly convex, coarsely punctate and pubescent; base slightly depressed. Scutellum triangular, apical margin slightly emarginate, finely punctate and pubescent.

**Elytra**: EL/EW = 1.4–1.6; elytral humeri slightly projecting antero-laterally, humeral groove shallow; lateral margin gradually expanding from anterior to posterior, the elytra widest behind the middle, apex rounded; disc slightly convex, coarsely punctate and pubescent; elytral base coarsely punctate and pubescent; suture with one row of punctures and pubescence; epipleura narrow, with two rows of punctures and pubescence at base and one row at apex.

**Abdomen and legs**: underside sparsely punctate and pubescent. Legs moderately long, femora robust. Pygidium moderately long, apical portion exposed, punctate, and pubescent. Apical margin of last abdominal ventrite slightly concave in female, slightly prominent in male. Median lobe strongly sclerotized, slender, dorso-central portion membranous with a tongue-shaped sclerite, curved in lateral view, lateral sides thin, subparallel, apex narrower than base, apical portion tongue-shaped, apex upward, sharp; median struts rod-shaped, widely separated from each other, 1.1 × as long as median lobe; basal piece rod-like, short, basal portion of tegmen Y-shaped, tegminal ring with base broad and gradually narrowed to apical portion, paramere sub-square, apical margin of paramere with dense setae; endophallus membranous, with paired granulated and sclerotized areas. Spiculum long, Y-shaped, apical portion tongue-shaped, weakly sclerotized. Ovipositor moderately long, sub-triangular, base broad, apex slightly narrowed, divided into two vaginal palpi, each side with two baculi, one baculus extending from the base of the ovipositor backwards, base slightly broad, apex slightly narrowed, length of two baculi proximal to each other at base; another baculus extended from the coxite to the middle of the ovipositor; the two baculi of each side proximal to each other but not fused; coxite strongly sclerotized, cylindrical, lateral margin apex with long setae; stylus short, small, distinct.

###### Distribution.

China (Beijing), Belarus, Czech Republic, Denmark, Estonia, Finland, the United Kingdom, Germany, Latvia, Lithuania, Norway, Poland, Sweden, Russia, Mongolia.

###### Host plant.

*Populus tremula* (Salicaceae).

###### Remarks.

A large number of this species was caught by Malaise trips set in Songshan for approximately four months. This species is similar to *Zeugophora cribrata* Chen, 1974, but differs in having a brown head and pronotum; the lateral tubercle of the pronotum is more prominent; the pronotum and elytra are sparsely punctate; with the apex of the median lobe gradually narrowed (head black, pronotum brown, slightly black; lateral tubercle of the pronotum less prominent; pronotum and elytra densely punctate; and the apex of the median lobe strongly constricted in *Z. cribrata*).

This species is also similar to *Zeugophora scutellaris* Suffrian, 1840 with regards to the shape of the lateral tubercle on the pronotum, but differs in having the head, pronotum, elytra and antennae yellowish brown; the pronotal disc is finely punctate while the elytra is sparsely punctate; the dorso-central portion of the median lobe has a slender sclerite, and the apex of the median lobe is slightly broad and blunt (head and pronotum reddish brown, elytra black, antennomeres 1–4 brown, antennomeres 5–11 black; pronotal disc coarsely punctate and elytra densely punctate; the dorso-central portion of the median lobe without a sclerite, and the apex slightly narrowed and sharp in *Z. scutellaris*).

#### Subgenus Pedrillia Westwood, 1864

##### Zeugophora (Pedrillia) euonymorum sp. nov.

Taxon classificationAnimaliaColeopteraMegalopodidae

5C3F8F46-7CAA-57B1-8A9B-BEA321FDB54C

http://zoobank.org/A9A6AB06-C0DE-45A2-B64F-5CE8D598820C

Figures 12–15 , 16–20 , 21–24 , 25–28

###### Specimens examined.

***Holotype*: **male, China, Ningxia, Jingyuan, Liupan Shan National Nature Reserve, Erlonghe Forest Farm, 35.33041°N, 106.35110°E / 2088 m, 2018.vii.29, Kaiqin Li coll., Kunming Institute of Zoology, Chinese Acad. Sci. / Holotype, Zeugophora (Pedrillia) euonymorum sp. nov., des. by K.Q. Li & H.B. Liang, 2020 [red label]. ***Paratypes*** (11 males and 12 females): 4 males and 7 females (1 male and 1 female in IZCAS), same data as holotype except Paratype Zeugophora (Pedrillia) euonymorum sp. nov., des. by K.Q. Li & H.B. Liang, 2020 / [yellow label]; 1 female, China, Ningxia, Jingyuan, Liupan Shan National Nature Reserve, Xiaonanchuan, 35.35788°N, 106.31659°E / 2021 m, 2018.vii.27, Xinpu Wang coll. Ningxia University / Paratype Zeugophora (Pedrillia) euonymorum sp. nov., des. by K.Q. Li & H.B. Liang, 2020 / [yellow label]; 3 males, 1 female, China, Ningxia, Jingyuan, Liupan Shan National Nature Reserve, Danangou, 35.48659°N, 106.27045°E / 2130 m, 2018.vii.28, Kaiqin Li coll. / Paratype Zeugophora (Pedrillia) euonymorum sp. nov., des. by K.Q. Li & H.B. Liang, 2020 / [yellow label]; 4 males and 3 females (1 male and 1 female in IZCAS) / China, Guizhou, Leishan, Leigong Shan National Nature Reserve, 26.38470°N, 108.20681°E / 2158 m, 2019.vii.16, Kaiqin Li coll. / Paratype Zeugophora (Pedrillia) euonymorum sp. nov., des. by K.Q. Li & H.B. Liang, 2020 / [yellow label].

**Figures 12–15.**
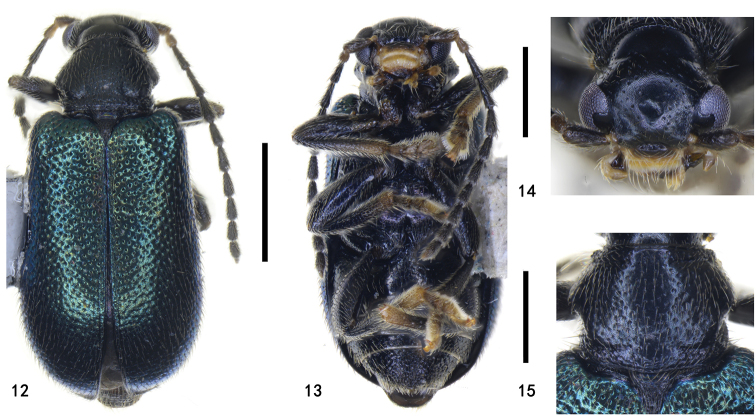
Holotype of Zeugophora (Pedrillia) euonymorum sp. nov., male **12** dorsal view **13** ventral view **14** head, anterior view **15** pronotum, dorsal view. Scale bars: 1.0 mm (**12, 13**); 0.5 mm (**14, 15**).

###### Diagnosis.

Head, pronotum, scutellum, underside black; elytra with dark blue or dark green metallic luster; antennae slender, exceeding half the length of body; lateral tubercle of pronotum gradually expanding from anterior to the middle and then constricted; median lobe with basal one third tubular, slightly flattened and curved in lateral view, apex triangular and blunt.

###### Description.

BL = 2.8–4.0 mm, BW = 1.3–2.0 mm. Head, pronotum, scutellum, underside black; elytra with dark blue or dark green metallic luster; mouthparts yellowish brown except mandible brown; antennae black or with antennomere 1 dark brown; legs black, sometimes basal portion of femora, apex of tibiae and most of tarsi yellow, or legs yellow.

**Head**: eyes prominent, inner margin with distinct canthus; vertex densely punctate and pubescent; occiput strongly constricted; frons sparsely punctate and pubescent, center with a shallow concave, lateral sides slightly depressed; clypeus rectangular, width 2 × length, punctate and pubescent laterally, separated from frons by deep fronto-clypeal suture; labrum rectangular, narrower than clypeus, anterior margin with punctures and pubescence; antennae slender, exceeding half the length of body, antennomere 1 long and swollen, antennomere 2 short, half as long as antennomere 1, antennomere 3 as long as 4, slightly longer than antennomere 1, antennomeres 5–10 as long as antennomere 1, antennomere 11 acute at apex; antennomeres 1–4 sparsely punctate and pubescent, antennomeres 5–11 densely punctate and pubescent.

**Thorax**: PW/PL = 1.3–1.5; anterior margin slightly flattened; posterior margin backwards medially; length of anterior margin nearly equal to posterior margin; anterior groove distinct laterally, obsolete medially; posterior groove deep laterally, shallow medially; anterior portion of lateral margin subparallel, then gradually expanding from anterior portion to middle, strongly constricted behind middle; lateral tubercle rounded; behind the lateral tubercle, an oblique groove extending to base portion; disc convex with punctures and pubescence, basal portion slightly depressed; basal portion of each side slightly prominent. Scutellum triangular, slightly emarginate at apex, densely punctate and pubescent.

**Elytra**: EL/EW = 1.4–1.6; elytral humeri projecting antero-laterally, humeral groove shallow, lateral of humeri densely punctate and pubescent; lateral margins slightly expanding from the base to the middle, widest behind the middle, apex rounded; disc slightly flattened, coarsely punctate and pubescent; suture with two rows of punctures and pubescence; epipleura narrow, two rows of punctures and pubescence at base and one row at apex.

**Abdomen and legs**: underside sparsely punctate and pubescent. Legs moderately long, femora robust, mid- and hind-tibiae slightly curved. Pygidium moderately long, apical portion exposed. Apical margin of last abdominal ventrite slightly prominent in male (apical margin of last abdominal ventrite nearly straight in female). Median lobe sclerotized, short and broad, slightly curved in lateral view, dorso-central portion membranous, basal one third tubular, lateral sides thick and parallel, apex triangular and blunt; median struts rod-shaped, widely separated from each other, approximately 2.1–2.3 × as long as median lobe; basal portion of tegmen Y-shaped, tegminal ring oval-shaped, paramere tongue-shaped, apical margin of paramere with setae; endophallus membranous, with paired granulated and weakly sclerotized area. Spiculum spoon-shaped, apical margin slightly emarginate. Ovipositor moderately long, sub-rectangular, base broad, apex slightly narrowed, divided into two vaginal palpi, each side with one baculus, extending from the base of the ovipositor backwards to the coxite, baculus sclerotized, base slightly broad, apex slightly narrowed, two baculi proximal to each other at base; coxite strongly sclerotized, cylindrical, lateral margin of apex with long setae; stylus, cylindrical, short, small and distinct.

**Figures 16–20.**
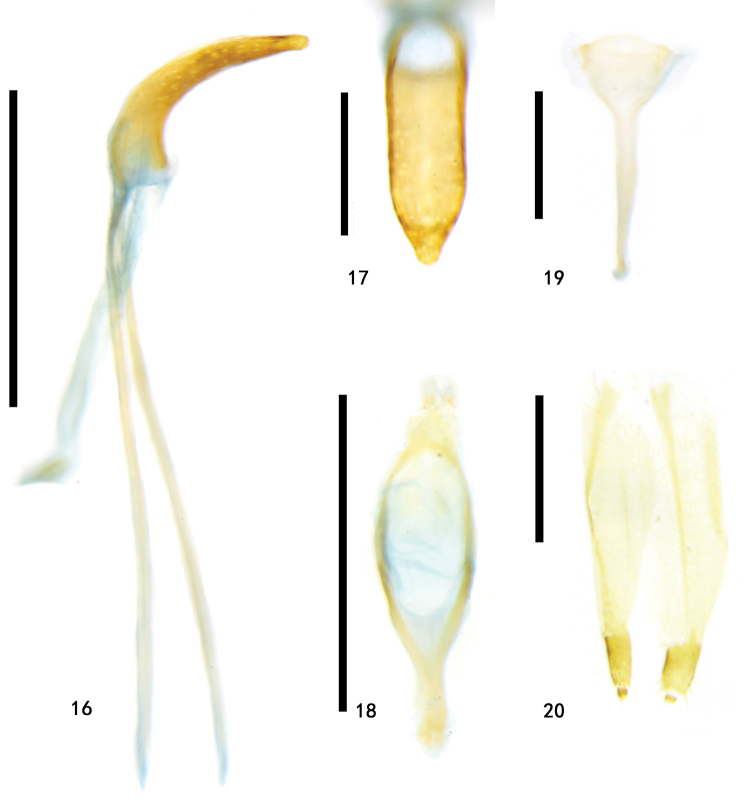
Types of Zeugophora (Pedrillia) euonymorum sp. nov. **16–19** male genitalia of holotype **16** median lobe and median struts, lateral view **17** median lobe, dorsal view **18** tegmen, dorsal view **19** spiculum, dorsal view **20** ovipositor of paratype, dorsal view. Scale bars: 0.5 mm (**16, 18**); 0.2 mm (**17, 19, 20**).

###### Distribution.

China (Ningxia, Guizhou).

###### Host plant.

*Euonymus elatus*, *Euonymus hamiltonianus* (Celastraceae).

###### Etymology.

The specific name *euonymorum* refers to the host plant genus *Euonymus* (Family Celastraceae).

###### Remarks.

This species is similar to *Zeugophora cyanea* Chen, 1974 in color, but differs in having longer antennae, the lateral tubercle of the pronotum gradually expanding from the anterior to the middle and then constricted, the lateral tubercle broader and bigger, the ratio of median struts / median lobe approximately 2.1–2.3, basal third of median lobe tubular with the apex of the median lobe broader and blunt, the tegminal ring oval-shaped, the paramere tongue-shaped, the spiculum long and Y-shaped and with the apex prominent (shorter antennae, lateral tubercle prominent on lateral middle of pronotum, anterior margin of pronotum slightly subparallel, lateral tubercle narrow and smaller, the ratio of median struts / median lobe approximately 1.1, median lobe dorso-central portion membranous and apical portion with a slender and weakly sclerotized sclerite, apex of median lobe narrower and sharper, tegminal ring gradually narrowed from base to apical portion, paramere sub-square, spiculum long and Y-shaped, and apex trifid in *Z. cyanea*).

The external male genitalia of this species are similar to those of Zeugophora (Pedrillia) annulata Baly, 1873, Zeugophora (Pedrillia) bicolor Kraatz, 1879, Zeugophora (Pedrillia) tricolor Chen & Pu, 1962, but differs from them in having a slightly flattened and less curved median lobe in the lateral view, with the apex of the median lobe shorter and slightly broader.

**Figures 21–24.**
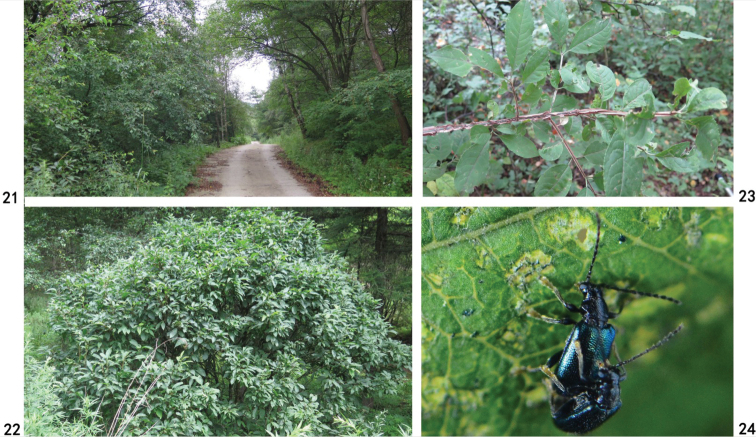
**21** Habitat of Zeugophora (Pedrillia) euonymorum sp. nov. (Ningxia, Liupan Shan) **22, 23** host plant of Zeugophora (Pedrillia) euonymorum (*Euonymus elatus*) **24** copulation.

**Figures 25–28.**
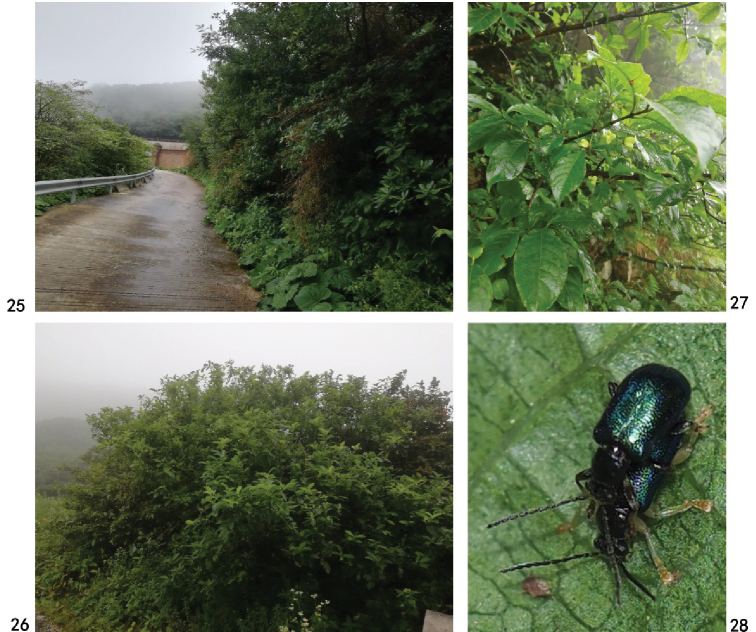
**25** Habitat of Zeugophora (Pedrillia) euonymorum sp. nov. (Guizhou, Leigong Shan) **26, 27** host plant of *euonymorum* sp. nov. (*Euonymus hamiltonianus*) **28** copulation.

##### Zeugophora (Pedrillia) flavithorax sp. nov.

Taxon classificationAnimaliaColeopteraMegalopodidae

2CF99E1C-40CC-5834-8EAA-CDF01462CBAE

http://zoobank.org/E25DF81C-67EF-410A-9855-AE2002ECE2B2

Figures 29–32 , 33–37 , 46–47

###### Specimens ecamined.

***Holotype***: male, China, Guizhou, Leishan, Leigong Shan National Nature Reserve, 26.36966°N, 108.18724°E / 1367 m, 2019.vii.14, Kaiqin Li coll. / Holotype, Zeugophora (Pedrillia) flavithorax sp. nov., des. by K.Q. Li & H.B. Liang, 2020 [red label]. ***Paratypes*** (1 male and 1 female): 1 female, same data as holotype but paratype Zeugophora (Pedrillia) flavithorax sp. nov., des. by K.Q. Li & H.B. Liang, 2020 [yellow label]; 1 male (IZCAS), China, Guizhou, Leishan, Leigong Shan National Nature Reserve, 26.36966°N, 108.18724°E / 1367 m, 2019.vii.15, Kaiqin Li coll. / Paratype Zeugophora (Pedrillia) flavithorax sp. nov., des. by K.Q. Li & H.B. Liang, 2020 [yellow label].

**Figures 29–32.**
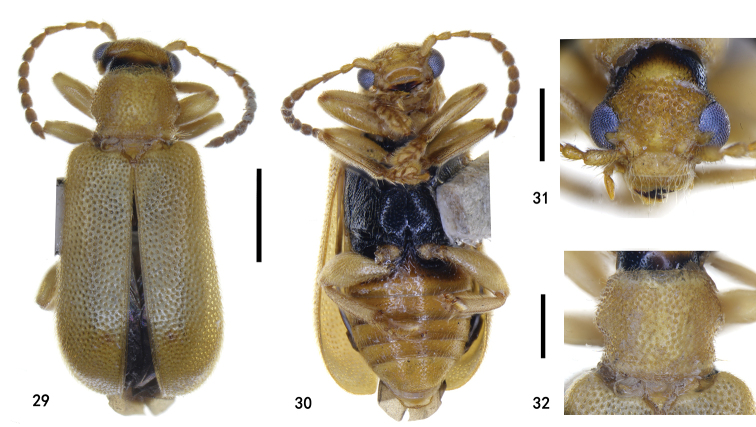
Holotype of Zeugophora (Pedrillia) flavithorax sp. nov., male **29** dorsal view **30** ventral view **31** head, anterior view **32** pronotum, dorsal view. Scale bars: 1.0 mm (**29, 30**); 0.5 mm (**31, 32**).

###### Diagnosis.

Head brown except occiput black, antennae, pronotum, elytra, prosternum, abdominal ventrites and legs brown, mesoventrite and metaventrite black, the pubescence of surfaces slightly yellow and transparent; median lobe short and broad, slightly curved in lateral view, lateral sides thin, apex slightly broad, middle of apical margin projecting and sharp; base of coxite with one elongate sclerotized area.

###### Description.

BL = 4.0–4.2 mm, BW = 1.6–1.8 mm. Head brown except occiput black, antennae, pronotum, elytra, prosternum, abdominal ventrites, and legs brown, mesoventrite and metaventrite black.

**Head**: eyes prominent, inner margin with distinct canthus; vertex coarsely and densely punctate and pubescent; occiput strongly constricted; frons finely punctate and pubescent; fronto-clypeal suture arching backwards centrally; clypeus rectangular, width 2.1 × that of length, anterior margin and lateral with punctures and pubescence; labrum rectangular, narrower than clypeus, center of anterior portion emarginate, anterior and lateral portion with punctures and pubescence; antennae extended to exceed the humeri, antennomere 1 long, anterior portion slightly swollen, antennomere 2 short, half-length of antennomere 1, antennomere 3 slightly shorter than antennomere 1, antennomere 4 as long as antennomere 1, antennomeres 5–11 slightly thick, antennomere 5 shorter than antennomere 4, antennomere 6 slightly shorter than antennomere 5, antennomeres 7–10 slightly shorter than antennomere 6, antennomere 11 as long as antennomere 6, acute at apex, antennomeres 1–4 sparely punctate and pubescent, antennomeres 5–11 densely punctate and pubescent.

**Thorax**: PW/PL = 1.2–1.3; anterior margin slightly flattened; posterior margin arching backwards in the center; length of anterior margin nearly equal to posterior margin; anterior and posterior groove shallow; lateral margin gradually expanding from anterior angle to the middle, then constricted, lateral tubercle projecting, narrow and blunt; disc slightly convex, coarsely punctate and pubescent. Scutellum triangular, apical margin slightly emarginate, sparsely punctate and pubescent.

**Elytra**: EL/EW = 1.5–1.7; elytral humeri slightly projecting antero-laterally, humeral groove shallow; lateral margin gradually expanding from anterior to posterior, the elytra widest behind the middle, apex rounded; disc slightly convex, weakly depressed at basal one third, coarsely punctate and pubescent; elytra base finely punctate and pubescent; suture with one or two rows of punctures and pubescence; epipleura narrow, two rows of punctures and pubescence at base and one row at apex.

**Abdomen and legs**: underside sparsely punctate and pubescent. Legs moderately long, femora robust. Pygidium moderately long, punctate, and pubescent, apical portion exposed; apical margin of last abdominal ventrite slightly prominent in male (slightly emarginate in female). Median lobe weakly sclerotized, short and broad, slightly curved in lateral view, dorso-central portion entirely membranous, lateral sides thin, apex slightly broad, middle of apical margin projecting and sharp; median struts rod-shaped, widely separated from each other, approximately 1.8 × as long as median lobe; base of tegmen V-shaped, tegminal ring sub-oval, paramere sub-trapezoid and apical margin slightly rounded, apical margin with long setae; endophallus membranous, with paired granulated and weakly sclerotized area. Spiculum long Y-shaped, apical margin slightly flattened. Ovipositor long, sub-rectangular, base slightly broader than apex, divided into two vaginal palpi, each side with one baculus, extending from the base of the ovipositor backwards to the coxite, baculus base slightly broad, apex slightly narrowed, sclerotized, two baculi proximal to each other at base; coxite strongly sclerotized, sub-rectangular, apical margin with setae, lateral sides of apical margin with stylus; stylus, cylindrical, small and distinct.

**Figures 33–37.**
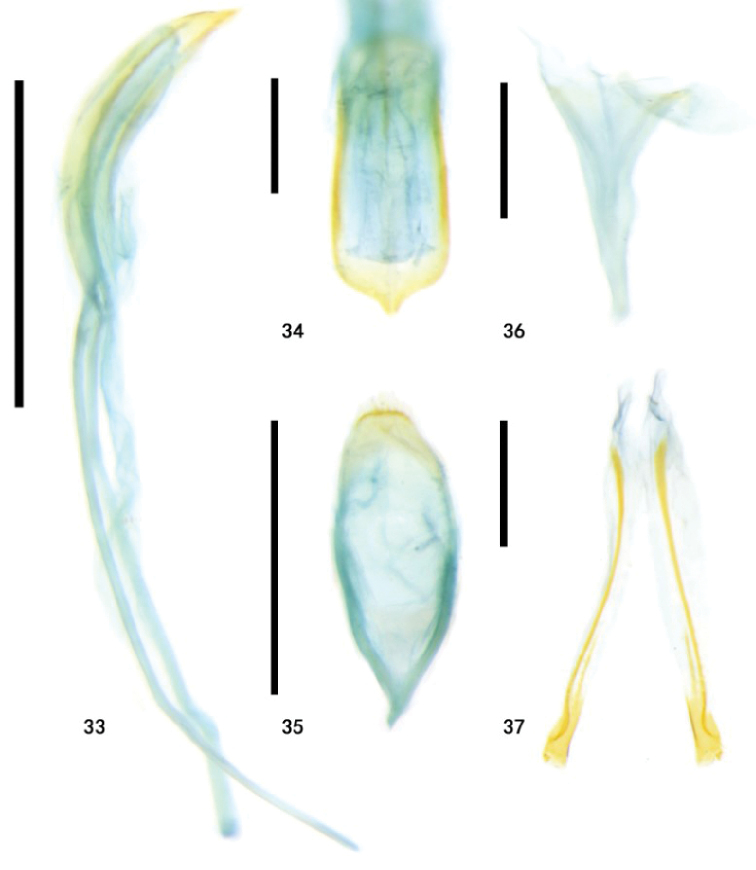
Types of Zeugophora (Pedrillia) flavithorax sp. nov., genitalia **33–36** male genitalia of holotype **33** median lobe and median struts, lateral view **34** median lobe, dorsal view **35** tegmen, dorsal view **36** spiculum, dorsal **37** ovipositor of paratype, dorsal view. Scale bars: 0.5 mm (**33, 35**); 0.2 mm (**34, 36, 37**).

###### Distribution.

China (Guizhou).

###### Host plant.

Symplocaceae.

###### Etymology.

The specific name *flavithorax* refers to the yellow color of the prothorax.

###### Remarks.

**Figures 38–41.**
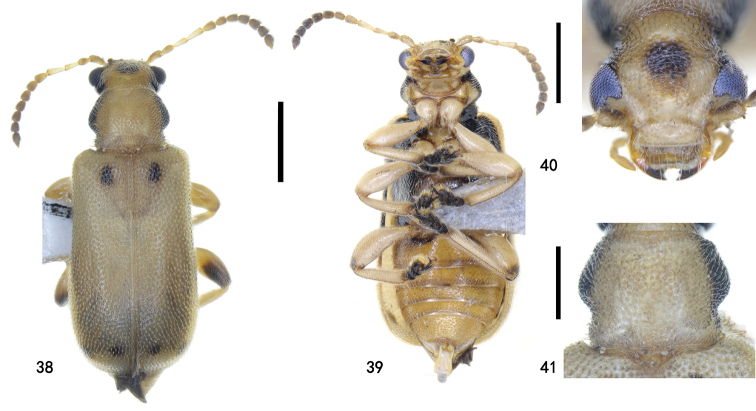
Zeugophora (Pedrillia) maculata (Chûjô) from Guizhou, female **38** dorsal view **39** ventral view **40** head, anterior view **41** pronotum, dorsal view. Scale bars: 1.0 mm (**38, 39**); 0.5 mm (**40, 41**).

**Figures 42–45.**
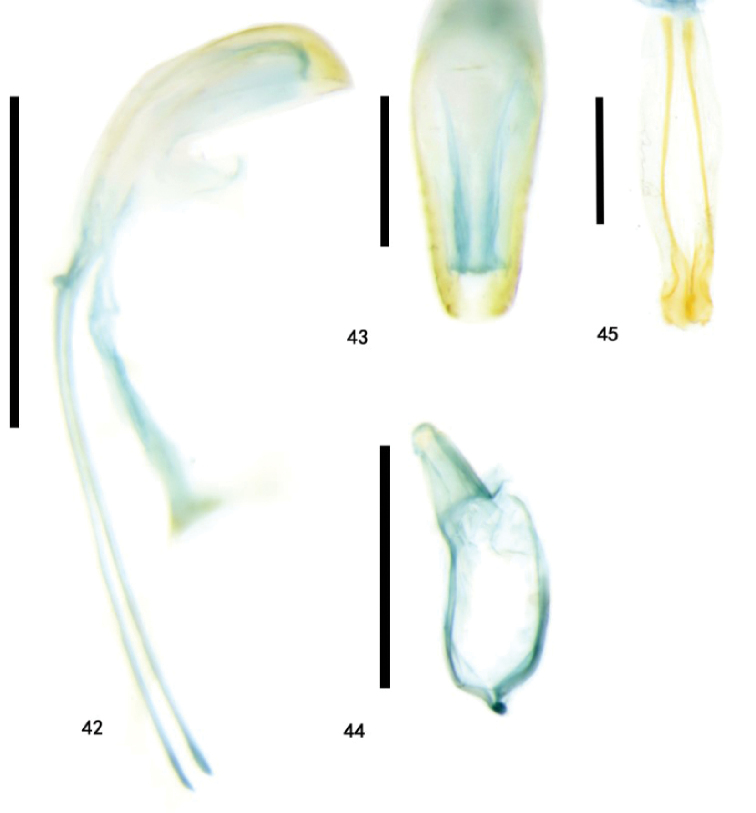
Genitalia of Zeugophora (Pedrillia) maculata (Chûjô) from Guizhou, genitalia **42–44** male genitalia **42** median lobe and median struts, lateral view **43** median lobe, dorsal view **44** tegmen, dorsal view **45** ovipositor, dorsal view. Scale bars: 0.5 mm (**42, 44**); 0.2 mm (**43, 45**).

This species is most similar to Zeugophora (Pedrillia) maculata (Chûjô, 1941) (Figs 38–41) in the shape of the external morphology and color, but differs in having the pronotum and elytra without a black spot, the pubescence of surfaces slightly yellow and transparent, the median lobe apex slightly broad and the apical margin projecting and sharp, paramere of tegmen sub-trapezoid, and the base of the coxite with one elongate sclerotized area (pronotum and elytra with a black spot, the pubescence of surfaces pale yellow, median lobe apex slightly narrowed and downward, apical margin slightly round without projecting, paramere of tegmen tongue-shaped, base of coxite without elongate sclerotized area in Z. (P.) maculata (Figs 38–45)).

**Figures 46–47.**
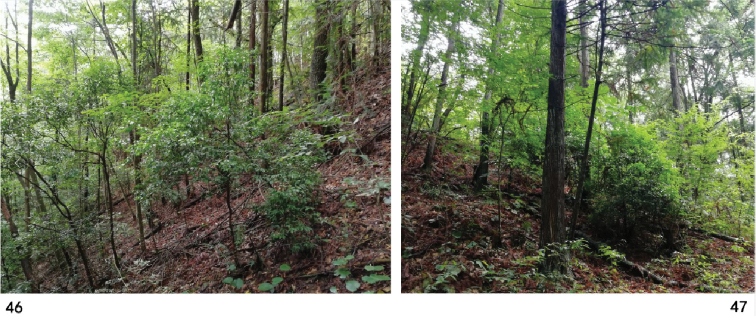
Habitat of Zeugophora (Pedrillia) flavithorax sp. nov. (Guizhou, Leigong Shan).

##### Zeugophora (Pedrillia) nigricollis

Taxon classificationAnimaliaColeopteraMegalopodidae

(Jacoby, 1885) new record

FB2CB5BF-9399-5B73-845F-EBA2E3450BC5

Figures 48–53 , 54–57

Pedrillia nigricollis Jacoby, 1885: 195. Type locality: Japan, Wada-tôge. Type
depository: NHMUK. Synonymized as Pedrillia bicolor Kraatz, 1879 by Kimoto 1986: 309.
Zeugophora (Pedrillia) nigricollis : Crowson, 1946: 95 [Japan]; Gressitt and Kimoto 1961: 24, 27; Chûjô 1954: 51; Chûjô and Kimoto 1961: 119; Kimoto 1964: 108.
Zeugophora (Pedrillia) bicolor : Kimoto, 1986: 309; Jolivet 1957: 12; Kimoto and Takizawa 1994: 6, 99, 267; An and Kwon 2002: 272; Silfverberg 2010: 334; Li and Liang 2018: 133.
Zeugophora bicolor : Gressitt, 1945: 139; An and Kwon 1995: 91–92; Takizawa 2006: 2; Rodríguez-Mirón 2018: 291.
Zeugophora nigricollis (Jacoby, 1885). Restored as a valid species by Takemoto 2019: 15–19.

###### Specimens examined.

**Type**: H. T. / Japan. G. Lewis. 1910–320. / *Pedrillia nigricollis* Jac.

**Non-types (KIZ)**. Two males, China, Ningxia, Jingyuan, Liupan Shan National Nature Reserve, Erlonghe Forest Farm, 35.33041°N, 106.35110°E, 2088 m, 2018.vii.27, Kaiqin Li coll.

**Figures 48–53.**
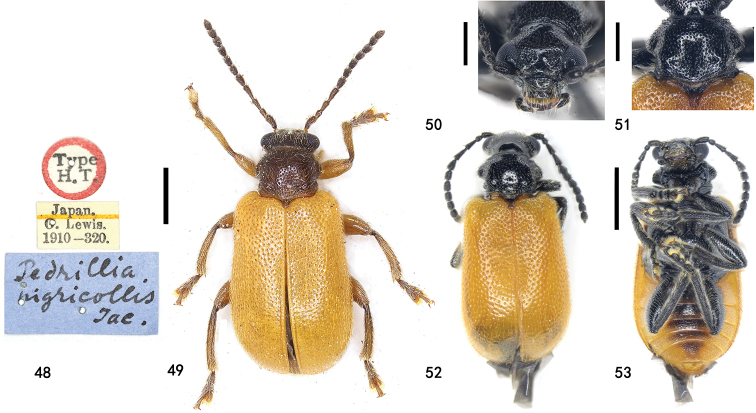
*Zeugophora nigricollis* Jacoby **48** holotype label **49** holotype dorsal view **50–53** specimens from Ningxia, male **50** head, anterior view **51** pronotum, dorsal view **52** dorsal view **53** ventral view. Scale bars: 1.0 mm (**49, 52, 53**); 0.5 mm (**50, 51**).

###### Diagnosis.

Elytra brown, head, antennae, pronotum, scutellum, legs, and underside black (except lateral sides of abdominal ventrites brown); median lobe slender, curved in lateral view, apex of median lobe triangular and blunt.

###### Description.

BL = 3.8–4.5 mm, BW = 1.9–2.2 mm. Elytra brown, head, antennae, pronotum, scutellum, legs, and underside black (except lateral sides of abdominal ventrites brown).

**Head**: eyes prominent, inner margin with distinct canthus; vertex coarsely and densely punctate and pubescent, the middle slightly smooth without puncture; occiput strongly constricted; frons densely punctate and pubescent; fronto-clypeal suture slightly arching backwards centrally; clypeus rectangular, width 2.1 × that of length, anterior and lateral margin densely punctate and pubescent; labrum rectangular, narrower than clypeus, center of anterior portion emarginate, anterior and lateral with punctures and pubescence; antennae extended to exceed the humeri, antennomere 1 long, anterior portion slightly swollen, antennomere 2 short, half-length of antennomere 1, antennomere 3 slightly shorter than antennomere 1, antennomere 4 as long as antennomere 1, antennomeres 5–11 slightly thick, antennomere 5 shorter than antennomere 3, antennomeres 6–10 slightly shorter than antennomere 5, antennomere 11 as long as antennomere 5, acute at apex, antennomeres 1–4 sparsely punctate and pubescent, antennomeres 5–11 densely punctate and pubescent.

**Thorax**: PW/PL = 1.5–1.6; anterior margin slightly flattened; length of anterior margin nearly equal to posterior margin; posterior margin arching backwards in the center; anterior groove distinct laterally, shallow medially; posterior groove shallow; lateral margin gradually expanding from anterior angle to the middle, then constricted, lateral tubercle broad and round; disc convex, densely punctate and pubescent, basal portion of each side slightly prominent; each anterior and posterior angle with two or three long setae. Scutellum triangular, apical margin flattened, sparsely punctate and pubescent.

**Elytra**: EL/EW = 1.3–1.5; elytral humeri projecting antero-laterally, humeral groove shallow; lateral margin gradually expanding from anterior to posterior, the elytra widest behind the middle, apex round; disc flattened, weakly depressed at basal one third, coarsely punctate and pubescent; elytra base densely punctate and pubescent; suture with one row of punctures and pubescence; epipleura narrow, with one row of punctures and pubescence.

**Abdomen and legs**: underside sparsely punctate and pubescent. Legs moderately long, femora robust, meso- and meta-tibia slightly curved. Pygidium moderately long, punctate, and pubescent; apical margin of last abdominal ventrite slightly prominent. Median lobe sclerotized, slender, curved in lateral view, lateral sides thick and parallel, dorso-central portion membranous with basal one third tubular, apex triangular and blunt, apex slightly leftward; median struts rod-shaped, widely separated from each other, approximately 2.6 × as long as median lobe; base of tegmen V-shaped, tegminal ring sub-oval, paramere long and triangular with apical margin with long setae; endophallus membranous, with paired granulated and weakly sclerotized area. Spiculum long and Y-shaped, with apical margin projecting.

###### Distribution.

China (Ningxia).

###### Host plant.

*Euonymus elatus* (Celastraceae) (Figs 22, 23).

###### Remarks.

Takemoto (2019) restored Zeugophora (Pedrillia) nigricollis Jacoby, 1885 to a valid species from the synonymy of Z. (*P.*) *bicolor* (Kraatz, 1879). This species is most similar to Zeugophora (Pedrillia) bicolor Kraatz, 1879 in having the black and brown color, but differs in having the pronotum entirely black, the median lobe less curved in the lateral view, the median lobe slender, with the apex of the median lobe triangular and blunt (the base of the pronotum is slightly brown, median lobe is more curved in the lateral view, median lobe slightly broader, and apex of median lobe narrower and sharper in Z. (P.) bicolor).

**Figures 54–57.**
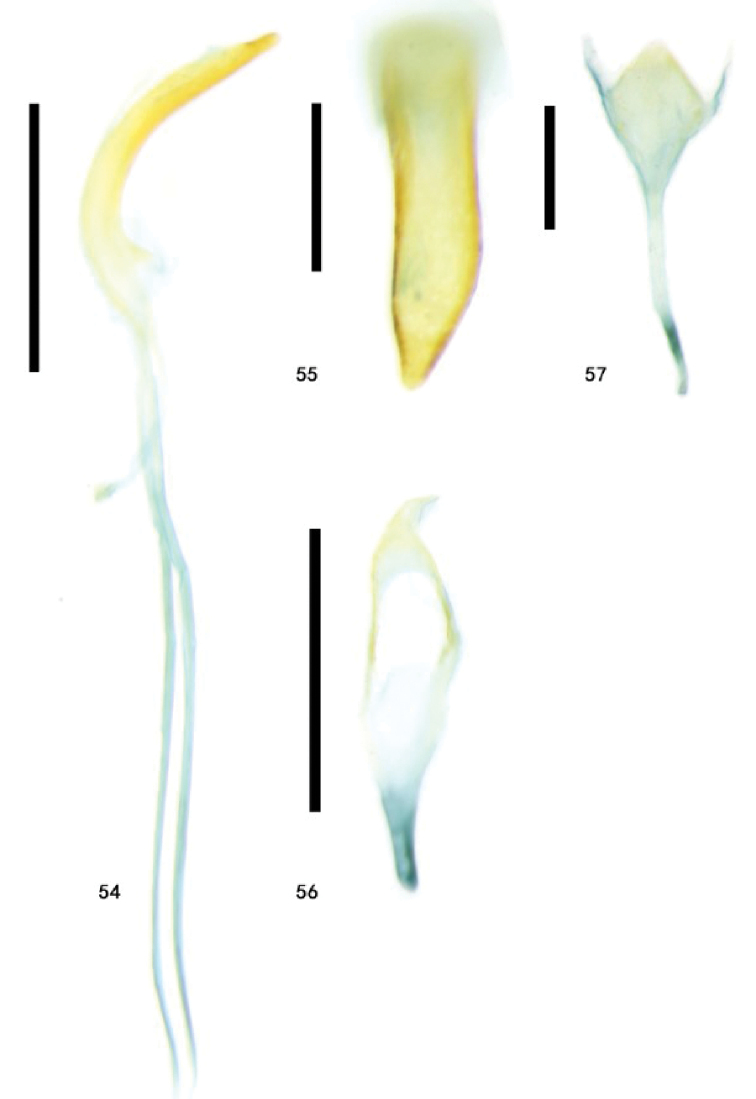
Genitalia of Zeugophora (Pedrillia) nigricollis Jacoby from Ningxia, male genitalia **54** median lobe and median struts, lateral view **55** median lobe, dorsal view **56** tegmen, dorsal view **57** spiculum, dorsal view. Scale bars: 0.5 mm (**54, 56**); 0.2 mm (**55, 57**).

##### Zeugophora (Pedrillia) trifasciata sp. nov.

Taxon classificationAnimaliaColeopteraMegalopodidae

16F1CAF7-3DFE-57AC-AAA2-029264FE6C55

http://zoobank.org/04D8AB26-4E47-45C3-8D75-3228A5E6A641

Figures 58–61 , 62–66

###### Specimens examined.

***Holotype* :** male, China, Yunnan, Ailaoshan, Xujiaba, 1986.viii.21 / Holotype, Zeugophora (Pedrillia) trifasciata sp. nov., des. by K.Q. Li & H.B. Liang, 2020 [red label]. ***Paratype***, 1 male, same data as holotype but 1986.x.31 / Paratype Zeugophora (Pedrillia) trifasciata sp. nov., des. by K.Q. Li & H.B. Liang, 2020 [yellow label].

**Figures 58–61.**
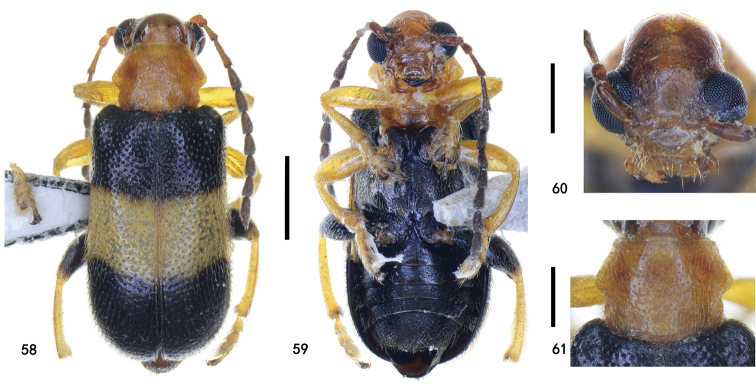
Holotype of Zeugophora (Pedrillia) trifasciata sp. nov., male **58** dorsal view **59** ventral view **60** head, anterior view **61** pronotum, dorsal view. Scale bars: 1.0 mm (**58, 59**); 0.5 mm (**60, 61**).

###### Diagnosis.

Head brown; antennae black, with basal four antennomeres brown; pronotum yellowish brown; scutellum dark brown; elytra dark blue with a pale yellow transverse band from epipleura to suture on the center; underside dark blue except prosternum and mesoventrite yellowish brown; legs yellowish brown except hind femur dark blue; antennae elongate, lateral sides of elytra sub-paralleled; dorso-central portion of the median lobe with one sclerite from base to apical portion.

###### Description.

BL = 4.3–4.4 mm, BW = 1.7–1.9 mm. Head brown; antennae black, with basal four antennomeres brown; pronotum yellowish brown; scutellum dark brown; elytra dark blue with a pale yellow transverse band from epipleura to suture on the center; underside dark blue except prosternum and mesoventrite yellowish brown; legs yellowish brown except hind femur dark blue.

**Head:** eyes prominent, inner margin with distinct canthus; center of vertex sparsely punctate and pubescent, lateral sides densely punctate and pubescent; occiput strongly constricted; frons finely punctate and pubescent, center slightly concave; clypeus rectangular, width 2.4 × that of length, central portion slightly prominent, punctate and pubescent laterally, separated from frons by deep fronto-clypeal suture; shallow oblique groove from inner margin of upper portion of eye to fronto-clypeal suture; labrum rectangular, slightly narrower than clypeus, anterior margin with punctures and pubescence; antennae slender, exceeding half the length of body, antennomere 1 long and swollen, antennomere 2 short, shorter than half the length of antennomere 1, antennomere 3 slightly shorter than antennomere 1, antennomere 4 subequal to antennomere 1, antennomere 5 equal to 6 in length, and two-thirds as long as antennomere 4, antennomeres 7–10 equal to each other in length and slightly shorter than antennomere 5, antennomere 11 slightly longer than antennomere 5 and apex acute, antennomeres 1–4 sparsely punctate and pubescent, antennomeres 5–11 densely punctate and pubescent.

**Thorax:**PW/PL = 1.5–1.6; anterior margin slightly flattened; posterior margin arching backwards medially; length of anterior margin nearly equal to posterior margin; anterior and posterior groove indistinct; lateral sides subparallel at anterior portion, then gradually expanding in the middle, strongly constricted posterior to the middle; lateral tubercle prominent and blunt; with a shallow oblique groove behind the lateral tubercle to basal portion; disc convex, with fine punctures and pubescence, basal portion slightly depressed; basal portion of each side slightly prominent. Scutellum trapezoid, slightly emarginate at apex, densely punctate and pubescent.

**Elytra:**EL/EW = 1.6–1.7; elongate, elytral humeri projecting antero-laterally, humeral groove somewhat deep; lateral sides subparallel; disc slightly flattened, base slightly prominent and posterior slightly depressed, base densely punctate and pubescent, apex coarsely punctate and pubescent; suture with one row of punctures and pubescence; epipleura narrow, base with two rows of punctures and pubescence, apex with one row of punctures and pubescence.

**Abdomen and legs:** underside sparsely punctate and pubescent. Legs moderately long, femora robust, mid- and hind-tibiae slightly curved. Pygidium moderately long, apex portion exposed. Last abdominal ventrite with apical margin slightly prominent. Median lobe flattened, slightly curved in lateral view, sides and apex sclerotized, dorso-central portion with one sclerite from base to apical portion, sides moderately thick, apical portion tongue-shaped, apex sharp; median struts rod-shaped, widely separated from each other, approximately 2.8 × as long as median lobe; basal portion of tegmen Y-shaped, tegminal ring sub-round, paramere trapezoid, apical margin of paramere slightly emarginate in middle, lateral sides with setae; endophallus membranous, with paired granulated and sclerotized area. Spiculum Y-shaped, apical margin trifid.

###### Distribution.

China (Yunnan).

###### Host plant.

Unknown.

###### Etymology.

The specific name *trifasciata* refers to the transverse bands on the elytra.

###### Remarks.

This species is similar to Zeugophora (Pedrillia) formosana Gressitt, 1945 in having the transverse bands on the elytra, but differs in having the antennae elongate, the lateral sides of the elytra subparallel, the base of the pronotum without a distinct transverse groove (antennae shorter, lateral sides of elytra gradually expanding posteriorly from the base, broadest behind the middle, and the base of the pronotum with a transverse groove in Z. (P.) formosana).

This species is also similar to Zeugophora (Pedrillia) pallidicinata Gressitt, 1945 in color, but differs in having the elytra elongate and the lateral sides subparallel (elytra broad and short, the lateral sides expanding posteriorly from the base, widest behind the middle in Z. (P.) pallidicinata).

**Figures 62–66.**
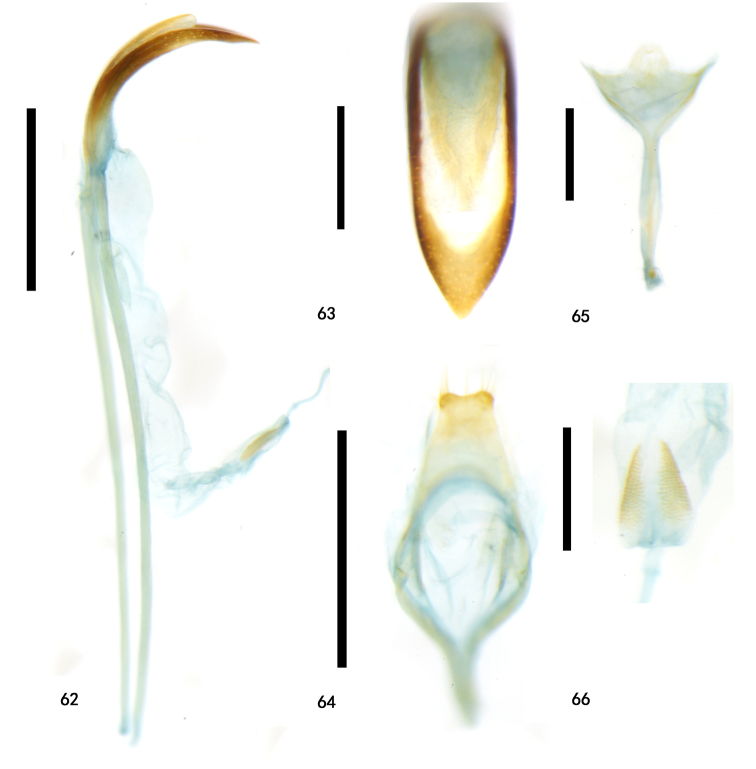
Holotype of Zeugophora (Pedrillia) trifasciata sp. nov., male genitalia **62** median lobe and median struts, lateral view **63** median lobe, dorsal view **64** tegmen, dorsal view **65** spiculum, dorsal view **66** sclerotized area of endophallus. Scale bars: 0.5 mm (**62, 64**); 0.2 mm (**63, 65, 66**).

The median lobe of this species is distinct with the dorsal portion of the median lobe having a long triangular sclerite (Figs 62, 63).

##### Zeugophora (Pedrillia) yuae sp. nov.

Taxon classificationAnimaliaColeopteraMegalopodidae

5A370F81-E735-5DFD-8340-2872B0460850

http://zoobank.org/92BC423A-5713-4D74-856B-F08CA4B0C047

Figures 67–70 , 71–75 , 76–79

###### Specimens examined.

***Holotype* :** male, China, Yunnan, Xishuangbanna, Nabanhe National Nature Reserve, Dayangdi, 22.24610°N, 100.60303°E / 911 m, 2019.v.10, Kaiqin Li coll. / Holotype, Zeugophora (Pedrillia) yuae sp. nov., des. by K.Q. Li & H.B. Liang, 2020 [red label]. ***Paratypes*** (4 males and 3 females): 1 male and 1 female (1 male in IZCAS), same data as holotype but paratype Zeugophora (Pedrillia) yuae sp. nov., des. by K.Q. Li & H.B. Liang, 2020 / [yellow label]; 3 males and 2 females (1 female in IZCAS), China, Yunnan, Xishuangbanna, Nabanhe National Nature Reserve, Dayangdi, 22.24610°N, 100.60303°E / 911 m, 2019.v.11, Kaiqin Li coll. / Paratype Zeugophora (Pedrillia) yuae sp. nov., des. by K.Q. Li & H.B. Liang, 2020 / [yellow label].

**Figures 67–70.**
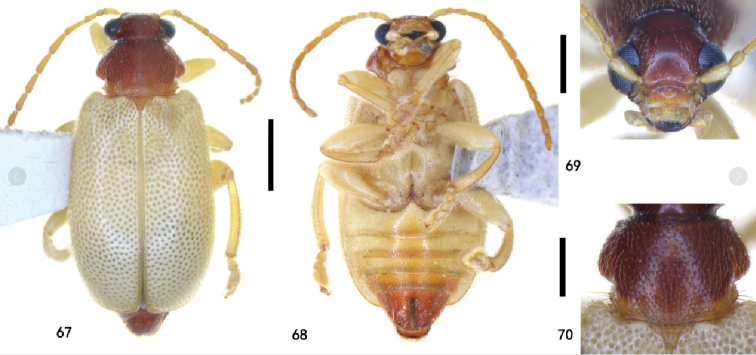
Holotype of Zeugophora (Pedrillia) yuae sp. nov., male **67** dorsal view **68** ventral view **69** head, anterior view **70** pronotum, dorsal view. Scale bars: 1.0 mm (**67, 68**); 0.5 mm (**69, 70**).

###### Diagnosis.

Head, prothorax, pygidium, last abdominal ventrite reddish brown; antennae and scutellum brown; elytra, mesoventrite, metaventrite, most portion of abdominal ventrites, legs pale brown; pubescence pale brown; antennae slender, extended to half the length of body; median lobe slender, slightly curved in lateral view, dorso-central portion membranous with basal one half tubular and other portion flattened, apex triangular, blunt.

###### Description.

BL = 3.3–4.7 mm, BW = 1.6–2.2 mm. Head, prothorax, pygidium, last abdominal ventrite reddish brown; antennae and scutellum brown; elytra, mesoventrite, metaventrite, most portion of abdominal ventrites, legs pale brown; pubescence pale brown.

**Head:** eyes prominent, inner margin with distinct canthus; vertex smooth, lateral sides near eyes finely and sparsely punctate and pubescent; occiput strongly constricted; frons finely punctate and pubescent; fronto-clypeal suture slightly arching backwards; clypeus rectangular, width 2.2 × that of length, anterior and lateral margin with punctures and pubescence; labrum rectangular, slightly narrower than clypeus, center of anterior portion emarginate, anterior and lateral portion with punctures and pubescence; antennae slender, extending to half the length of body, antennomere 1 long, anterior portion slightly swollen, antennomere 2 short, less than half the length of antennomere 1, antennomere 3 slightly shorter than antennomere 1, antennomere 4 as long as antennomere 1, antennomeres 5–11 slightly thick, antennomere 5 as long as antennomere 4, antennomeres 6–8 slightly shorter than antennomere 5, antennomere 9 as long as antennomere 10 and slightly shorter than antennomere 8, antennomere 11 as long as antennomere 10, acute at apex, antennomeres 1–4 sparsely punctate and pubescent, antennomeres 5–11 densely punctate and pubescent.

**Thorax:**PW/PL = 1.5–1.7; anterior margin slightly flattened; posterior margin arching backwards centrally; length of anterior margin nearly equal to posterior margin; anterior groove shallow laterally, indistinct medially; posterior groove deep laterally, shallow medially; lateral margin gradually expanding from anterior angle to beyond the middle, then constricted, lateral tubercle broad and round; disc slightly convex, sparsely punctate and pubescent, each side of the basal portion slightly depressed. Scutellum trapezoid, apical margin slightly flattened, sparsely punctate and pubescent.

**Elytra:**EL/EW = 1.3–1.5; elytral humeri projecting antero-laterally, humeral groove shallow; lateral margin gradually expanding from anterior to posterior, the elytra widest behind the middle, apex rounded; disc slightly convex, weakly depressed at basal one third, coarsely punctate and pubescent; base of elytra finely punctate and pubescent; suture with one row of punctures and pubescence; epipleura narrow, two rows of punctures and pubescence at base and one row at apex.

**Abdomen and legs:** underside sparsely punctate and pubescent. Legs moderately long, femora robust, mid- and hind-tibiae slightly curved; the mid-femur with a small ventral tooth in the male (without a small ventral tooth in the female). Pygidium long, with punctures and pubescence, most exposed, apical margin slightly truncated; last abdominal ventrite center with one ridge, apical margin slightly prominent in male (last abdominal ventrite center without ridge, apical margin slightly straight in female). Median lobe sclerotized, slender, slightly curved in lateral view, dorso-central portion membranous with basal half tubular and other half flattened, apex triangular, blunt; median struts rod-shaped, widely separated from each other, approximately 3.5 × as long as median lobe; base of tegmen V-shaped, tegminal ring slightly subparallel, parameres tongue-shaped, apical margin with long setae; endophallus membranous, with paired granulated and weakly sclerotized area. Spiculum long, spoon-shaped. Ovipositor long, sub-rectangular, base broad, apex slightly narrowed, divided into two vaginal palpi, each side with one baculus, extending from the base of the ovipositor backwards to the coxite, baculus base slightly broad, apex slightly narrowed, base sclerotized and gradually less sclerotized from base to apex, two baculi proximal to each other at base; coxite strongly sclerotized, cylindrical, apical margin with long setae; stylus, cylindrical, small and distinct.

**Figures 71–75.**
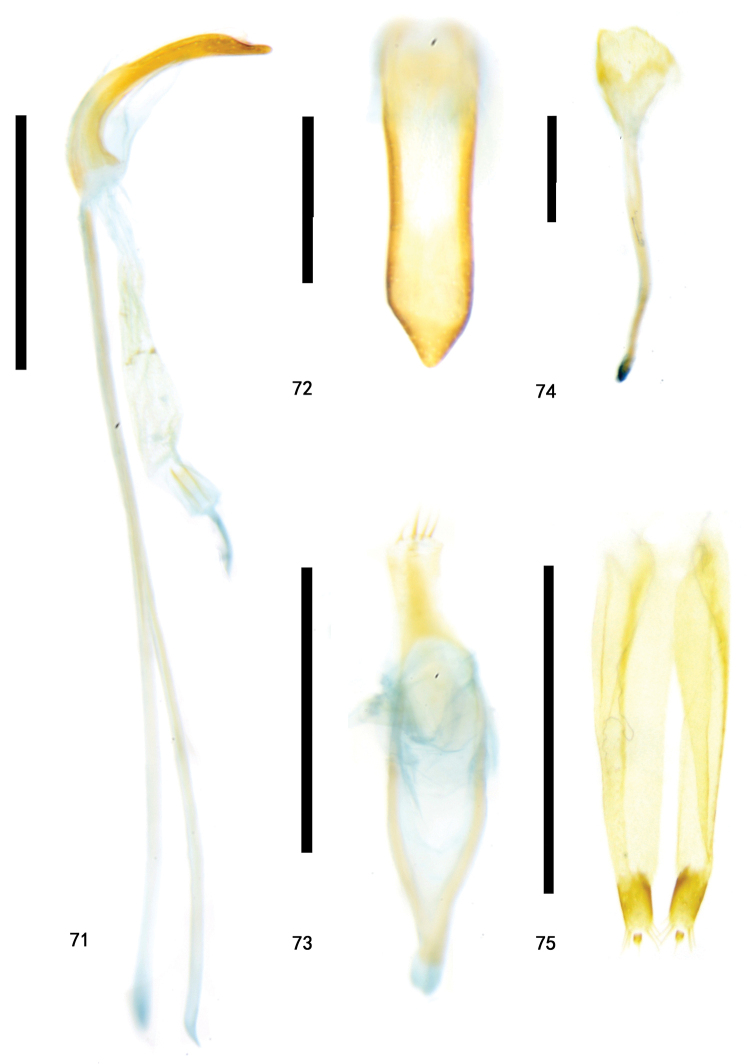
Types of Zeugophora (Pedrillia) yuae sp. nov., genitalia **71–74** male genitalia of holotype **71** median lobe and median struts, lateral view **72** median lobe, dorsal view **73** tegmen, dorsal view **74** spiculum, dorsal **75** ovipositor of paratype, dorsal view. Scale bars: 0.5 mm (**71, 73**); 0.2 mm (**72, 74, 75**).

###### Distribution.

China (Yunnan).

###### Host plant.

*Vitex quinata* (Lamiaceae) (Figs 77, 78).

**Figures 76–79.**
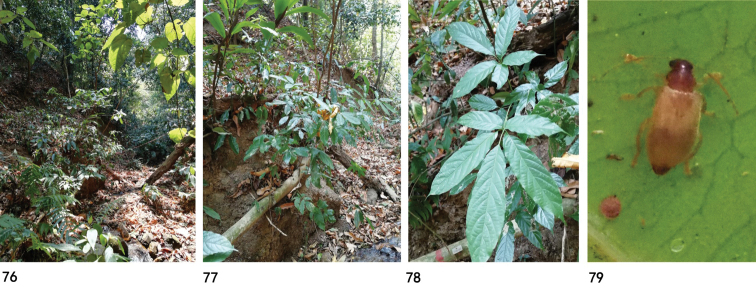
Habitat, host plant and adult of Zeugophora (Pedrillia) yuae sp. nov. **76** habitat (Yunnan, Nabanhe) **77–78** host plant (*Vitex quinata*) **79** dorsal view.

###### Etymology.

The specific name *yuae* is proposed in memory of Professor Peiyu Yu, who has greatly contributed to the taxonomy of the Chinese Megalopodidae.

###### Remarks.

This species is similar to Zeugophora (Pedrillia) tricolor Chen & Pu, 1962 in having a reddish-brown pronotum and pale brown elytra, but can differ from it in having the head reddish brown, antennae brown and slender, the base of the pronotum without a distinct tubercle, the median lobe less curved in the lateral view, the apex of the median lobe slightly broader and blunt, and the ratio of median struts / median lobe approximately 3.5 (head black, antennomeres 1–4 pale brown, antennomeres 5–11 black, antennae shorter and slightly robust, the base of the pronotum with a slight tubercle, the median lobe more curved in the lateral view, the apex of the median lobe slightly narrower and sharper, and the ratio of median struts / median lobe approximately 2.2 in *Z. tricolor*).

## Supplementary Material

XML Treatment for Zeugophora (Zeugophora) turneri

XML Treatment for Zeugophora (Pedrillia) euonymorum

XML Treatment for Zeugophora (Pedrillia) flavithorax

XML Treatment for Zeugophora (Pedrillia) nigricollis

XML Treatment for Zeugophora (Pedrillia) trifasciata

XML Treatment for Zeugophora (Pedrillia) yuae
